# Medical Society of the County of Albany

**Published:** 1872-03

**Authors:** James S. Bailey


BUFFALO

### Medical and Surgical Journal.

VOL XI MARCH, 1872. No. 8.

### Original Communications.

:o:

ART. I.—*Medical Society of the County of Albany. Semi-Monthly Meeting, Feb. 21st,* 1872. *Reported by* JAMES S. BAILEY, M.D.

DR. JOSEPH LEWI, President, in the Chair.

After the reading and approval of the minutes of the last meeting, the names of the following gentlemen were proposed for membership, viz. :—

Drs. Caleb Lyon, Stephen A. Ingham, E. J. Fisk and R. H. Starkweather.

Dr. R. H. SABIN reported the following case:

February 19th, 1872 was called to see Mrs. F., aged 18 years. She was suffering great pain in the head and back, with high fever, tongue swolen and covered with a dark brown colored fir, thirst was great, and the inside of the lips was very sore and bleeding upon the slightest touch. She vomited every thing that was taken into the stomach. The conjunctiva was much congested, the skin hot and scarlet. She was seven months advanced in pregnancy, urine scanty and passed with difficulty. Upon the morning of the 20th had slept about two hours. Symptoms much the same as the day previous ; water was not passed voluntarily so Dr. S. resorted to catheterization. The redness of the skin had somewhat subsided, there was no eruption. Stomach still rejected every thing taken Os uteri somewhat dilated and slight hemorrhage. The fœtal heart could not be heard. Dr. Quackenbush was called in the evening. Symptoms very much the same. She was gradually sinking, and died at 9 P.M. Shortly before death she had spasms when purpuric spots appeared upon various parts of the body. The matter vomited was nearly black.

Dr. Sabin also reported a case of *Uterine Hemorrhage.* Mrs. M. aged 48, usually menstruated regularly. Two years ago had had an attack of uterine hemorrhage. Her youngest child was 20 years old; three years after his birth she had a three months miscarriage. She bled profusely for several days. The per Sulph. Ferri was used per vagina successfully though she sank rapidly from nervous exhaustion, but finely made a good recovery though slow. The hemorrhage this time commenced with her menstrual flow. As she did not usually bear medicine well, he again used the Ferri per Vagina, but owing to the lack of fibrine in the blood it did not coagulate it. Upon the 9th Dr. Quackenbush counciled with him and thought the hemorrhage came from the uterine walls. Dr. Seymor counciled also. They then used Elixir Vitriol, Sugar of Lead and Ergot, but she received more relief by use of the per Sulph Ferri applied to pledgets of lint used as plugs to the vagina. She again sank with nervous prostration. Vomited much which seemed to threaten her life. Her heart beat very feebly, Dr. S. then resorted to Ferri and beef tea injections, with 20 grs. of the bromide of ammonium. Afterwards used the carb. of ammonia, with water and milk in the same way. She is now able to sit up. He is now using oxygen gas with marked improvement. She consumes four gallons per day, one-half in the morning and the rest in the afternoon.

Dr. Beckett objected to the treatment; said he would have plugged the vagina and then given Ergot, and if the stomach would not have tolerated opium, he would have used the hypodermic injections of morphia in this case. With this treatment the hemorrhage would have ceased in three days, instead of lasting three weeks. Remarks were made by other gentlemen, when the President announced that it was time for recess and refreshments; after which,

Dr. Estes H. Davis read a paper upon *Inflammatory Phthisis.* Dr. D. remarked that every disease to which the human system is liable, however little the suffering that may attend it, and although free from all danger to life, is still worthy of the attention and consideration of the medical practitioner.

Some diseases have interest from the rarity even of their occurrence, so that but few of any period have the opportunity to observe them ; while others, from their wide spread prevalence and frequent occurrence, constitute a large share of the cases upon which he is ordinarily called to attend. Others from their frequent or general fatality, and prevailing as extended, and devastating epidemics have, like Small Pox, and the plague of the past, and like Asiatic or epidemic cholera, and spinal meningites, or spotted fever of the present day forced themselves upon the highest attention and stimulated the most exalted energy. To stay and mitigate their terrible devastation, have put in requisition all the known resources of our art. Inflammatory Phthisis, or Phthisis Pulmonalis has not, like the tornado of a vast epidemic, swept from existence its thousands in a day ; but like a mighty avalanche, it has moved forward with resistless force, and in its onward and unstaid course from age to age it has crushed out the life and buried in the tomb its tens of thousands of our race ; and while the world has looked anxiously to the profession of medicine for help and succor from this terrible malady, they have for the most part been able to give in return only that which embraces within itself all other ills —the word hopeless. It appears from the statistics given by Drs. Curtis, Haywood and Emerson, that the mortality from consumption in the cities of Boston, New York and Philadelphia, for a period varying from twenty to thirty years was from about fourteen to over eighteen per cent. of all the deaths that occurred in them. In Wood's Practice of Medicine we find this language : "This disease is probably the greatest existing scourge of the human race, at least in the northern and middle latitudes. It will not be deviating far from the truth to state that it causes one sixth or one seventh of all the deaths north of the tropics." It will not excite surprise that in former times when prejudice prevented frequent examinations of the body after death, and the progress of knowledge with the present appliances of our art not then enabling the physician to detect the morbid changes that were progressing during life, or frequently note the nature of the changes that were left after death, that the essential character of this malady was not then well understood. But with all our advancement in medical knowledge and improvements in diagnosis, especially in diseases of the lungs, I think I may safely venture the opinion that we are still unsettled in the pathology of Phthisis, its causes, and many of the pathological changes that it involves. Since the researches and writings of Laennec and Louis it has generally been conceded that their teachings were correct, and that Phthisis essentially consisted of a depraved state of the blood, associated with a morbid condition of the lungs, by which was effused into them an unhealthy lymph constituting in the language of William's Pathology cacoplastic and aplastic plasma, and agreeing with the miliary gray gelatinous and crude or yellow tubercle of Louis and Laennec. The deposition of this plasma is unattended, according to these authors, by any inflammatory action in the lungs, either as a necessary cause or a consequence of their development, and in four-fifths of patients fever does not become associated with tuberculosis till a more or less advanced stage. Lebert, who takes this view of the pathology of Phthisis, says it is important to note that, as a rule, no cause for chronic pulmonary tuberculosis can be found, and the popular saying that a neglected catarrh leads to consumption, is false. These opinions would seem to have very generally prevailed with the profession ever since their advocacy by these celebrated authors. But there have been some medical men, and the number seems to be increasing, who, without denying the existence of some such cases of phthisis, still believe that they constitute but a small proportion of the whole number, and that in most cases inflammation is the very foundation of the disease, and the change of structure but the result of inflammatory processes. That all forms of pneumonia may produce it, but especially the chronic interstitial. If this theory be true, in so far must the other prove pernicious in practice, for, as Niemeyer asserts, in very many cases there is not a single tubercle in phthisical lungs. It would seem that the physical signs as elicited by auscultation and percussion are essentially the same in tuberculosis, caseous infiltration, abscess and red and gray hepatization of the lungs, and what is true of one is, in the main, common to all.

Mr. Skoda, in his work on auscultation and percussion, says, "The auscultatory and percussion signs of indurated lungs are the same as those of hepatization, and what has been said of their modification in hepatization holds good in cases of induration." I was called to make a *post mortem* examination of the lungs of Mrs. S. Two of her sisters had died within a short time with Phthisis. I had not seen her during her sickness, but was informed she exhibited the usual symptoms of that disease, which terminated her life within three months from the commencement of her illness.

I found the left lung studded with small abcesses, especially the upper half, varying in size from a small pea to that of a hickory nut, and a few still larger; some of them communicated with each other by small openings, and a few with bronchial tubes, others were separate and entire. The substance inclosing the pus, and lying between the collections, extending some distance from their walls was a consolidated mass, having none of the appearance of lung tissue. Some of the lower portions of the lung were infiltrated with pus. The right lung was in a similar condition, but not so far advanced in structural change.

I did not find a tubercle in either lung, but inflammatory action was plainly apparent in both. This case would seem to be one of acute inflammatory Phthisis or quick consumption, with no tuberculous deposits, but exhibiting the caseous infiltration of Niemeyer as a result of inflammatory action.

I was called to see Mr. A. a farmer, in company with his attending physician, in the last stage of phthisis, and received from himself the circumstances attending the origin of his sickness. While plowing, two years before, with a spirited pair of horses, the plow came violently in contact with some obstruction by which the handle was thrown backwards with great force, striking him a severe blow on the chest over the sternum. He felt that he was quite severely hurt, but still was able for a time to continue his labor on the farm. He continued however to feel pain and soreness in the region of the injury, which gradually extended through the chest, followed by cough, purulent expectoration, and an ultimate development of all the symptoms of a confirmed phthisis, which terminated fatally in about two years from the time of his receiving the injury. After his death, I was called by his brother, who was himself an intelligent physician, to make an examination of the lungs. I found a large portion of them completely disorganized, and not showing any appearance of lung structure. The disorganization consisted of numerous small abcesses from the size of a shot to that of a small filbert, lying in a mass of induration, of which some portions were firm, while others constituted a soft solid homeogenious mass of low organization. The cacoplastic plasma of Williams' and the caseous infiltration of Niemeyer exemplifying the language of Carpenter that, as the ultimate tendency of inflammation is to produce the disintegration of the part so the ultimate tendency of the fibrinous exudation is to keep its elements together.

As I did not find a tubercle in either lung, and as this case was clearly inflammatory *ab Initio*, I could but regard it as a marked and interesting exhibition of inflammatory phthisis. The microscopic observations of Gerber, Gulliver, Addison, Watt and others, are a remarkable confirmation of the opinion that tubercular matter, pus and coagulable lymph, are but varieties of the same albuminous matter that exists in the blood, and that these products, to which may be added the caseous infiltration of Niemeyer, are, but the result of inflammation, and the materials of which new membranes, textures and deposits are formed, and present every variety of plasticity from that of perfect cicatrices and false membranes down to that of yellow tuberculous matter. In short, that both induration and softening occur as the result of inflammation, and the circumstances which determine suppuration are, first, a certain intensity of the inflammation, and second, a peculiar condition of the blood, but, if the morbid action be of a lower kind the nutrition of the natural texture is only impaired, not arrested, and from the increased deposition of solid matter, induration or consolidation takes place. If we admit these conclusions to be true, in connection with the *post mortem* appearances of many cases of phthisical lungs, proving the absence of tuberculosis, and that these, with many cases having tuberculous deposits have their origin in some form of inflammatory action, then the evidence is forced upon us that non-inflammatory tubercular phthisis is the exception, while that form of phthisis resulting from pre-existing inflammation is by far the more prevalent and common.

These organic changes of the lungs in phthisis must be admitted are for the most part without the pale of successful medication, but, if always, or even generally, they are preceded by inflammatory action as a cause, then, so far as all forms of inflammation modified by any changes in the circulating fluid are ameniable to medicine, so far we may reasonably hope that our efforts will be rewarded with success by early, persistent and judicious treatment.

After a lengthy discussion upon Dr. Davis's paper by every member present the Society adjourned.

